# Underwater endoscopic submucosal dissection performed under general anesthesia for the safe resection of superficial esophageal squamous cell carcinoma with ductal involvement

**DOI:** 10.1055/a-2277-0748

**Published:** 2024-03-14

**Authors:** Yusuke Takahashi, Kotaro Shibagaki, Satoshi Kotani, Kenichi Kishimoto, Shinsaku Tanaka, Norihisa Ishimura, Shunji Ishihara

**Affiliations:** 1 175764 Gastroenterology, Shimane University Faculty of Medicine, Izumo, Japan; 2 653239 Endoscopy, Shimane University Hospital, Izumo, Japan

A superficial esophageal squamous cell carcinoma (ESCC) will sometimes extend into the ducts of the submucosal esophageal glands (SEGs), so-called “ductal involvement,” with the frequency of this reported to be 21.3%–37.1% 1 2 . SEGs are generally ignored during an endoscopic submucosal dissection (ESD) procedure, although they should be considered because of the potential of ductal involvement. For digestive tumor cases, underwater endoscopic submucosal dissection (UESD) procedures can reportedly assist with the submucosal approach by raising the mucosa through buoyancy 3 ; however, this is not usually applied for superficial ESCC cases owing to the severe risk of aspiration. The results presented of UESD for superficial ESCCs with ductal involvement being performed with the patient under general anesthesia indicate good safety and a high level of resectability.

A man in his 60s underwent UESD under general anesthesia for two superficial ESCCs in the mid-esophagus ( Fig. 1 ; Video 1 ). An endoscope (GIF-H290T; Olympus Medical System Co., Tokyo, Japan), electrosurgical unit (VIO300 ERBE; Elektromedizin, Tübingen, Germany), and knife for UESD (DualKnife; Olympus Co.) were used. Submucosal dissection was performed using Endocut mode (60 W, effect 2) to limit damage to the proper muscle layer and SEGs. The lesion was prone to submersion by esophagogastric de-aeration. An endoscopic waterjet with saline solution was used for submucosal injection and to provide upward mucosal traction through buoyancy, resulting in a thickened submucosal layer that provided space for safe submucosal dissection. The submucosal layer under the SEGs was dissected, while SEGs close to the proper muscle layer were also safely removed through use of the waterjet and underwater condition ( Fig. 2 ). The lesions and all visible SEGs were completely resected with little muscular injury that could have caused esophageal stenosis 4 . Histologically, the lesions were diagnosed as superficial ESCCs limited to the lamina propria mucosae, with ductal involvement extending into the submucosal layer ( Fig. 3 and Fig. 4 ).

**Fig. 1:**
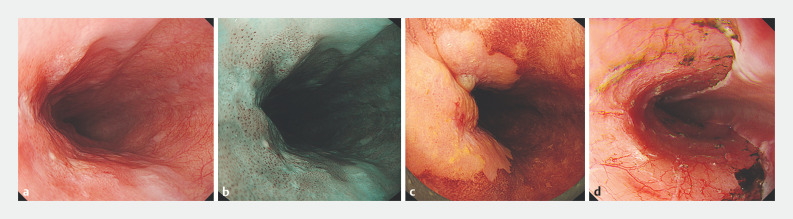
Endoscopic images of a superficial esophageal squamous cell carcinoma treated by underwater endoscopic submucosal dissection showing: **a** on a white-light image, a flat lesion with a reddish rough surface on the left side of the mid-esophagus; **b** on narrow-band imaging with low magnification, dot-like microvessels, suggesting intramucosal squamous cell carcinoma; **c** after iodine staining, the lesion extending horizontally to about half of the circumference; **d** the resected surface after completion of the procedure with scant muscular injury.

**Fig. 2:**
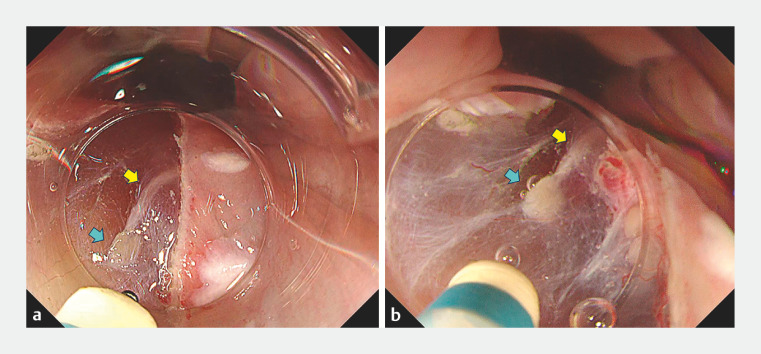
Endoscopic images of the resection of submucosal esophageal glands (SEGs) by underwater endoscopic submucosal dissection (UESD) with the patient under general anesthesia showing: **a** on a standard view during the ESD procedure, the SEG (blue arrow) and its duct (yellow arrow) in the submucosal layer, located close to the proper muscle layer; **b** with the use of the waterjet to create the underwater condition, the thickened submucosal layer, which raised the SEG up from the proper muscle layer, thereby allowing ESD to be safely performed under the SEG.

**Fig. 3:**
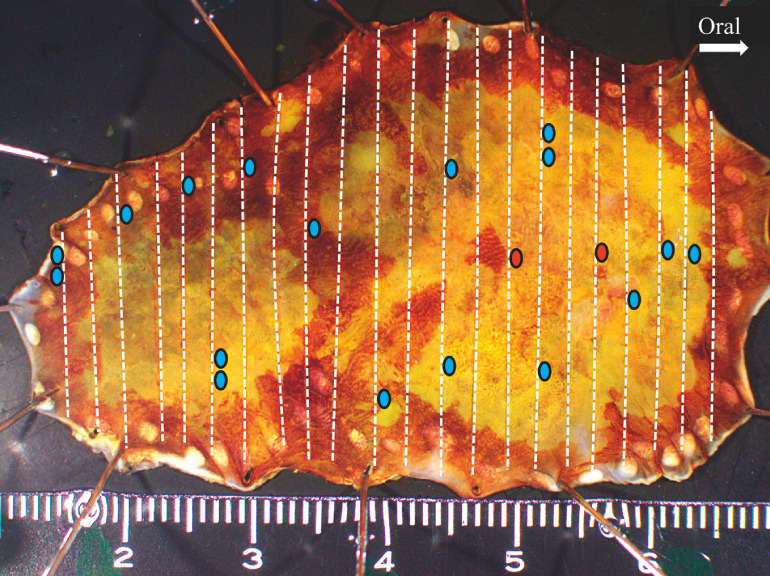
Photograph of the resected specimen stained with iodine showing two superficial esophageal squamous cell carcinomas that were completely resected. The white broken lines indicate the cutting lines and the many submucosal esophageal glands in the submucosal layer are indicated by blue ovals, and red ovals where ductal involvement of the gland was confirmed.

**Fig. 4:**
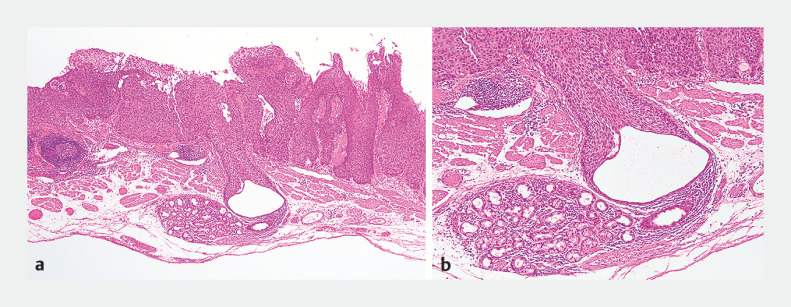
Histopathologic features of the superficial esophageal squamous cell carcinomas (ESCCs) with ductal involvement showing: **a** a superficial ESCC in the lamina propria mucosae that had invaded the duct leading to a submucosal esophageal gland; **b** a superficial ESCC partially located in the submucosal layer that showed ductal involvement, although the basement membrane of the duct was maintained – the cancer invasion depth was determined to be the lamina propria mucosae, suggesting a curative resection procedure 1 .

Underwater endoscopic submucosal dissection is performed with the patient under general anesthesia to safely remove submucosal esophageal glands, leading to the prevention of a positive vertical margin in a case of superficial esophageal squamous cell carcinoma with ductal invasion.Video 1

A UESD procedure performed under general anesthesia can help prevent a positive vertical margin in superficial ESCC cases with ductal involvement.

Endoscopy_UCTN_Code_TTT_1AO_2AG_3AD

## Notes

Endoscopy E-Videos https://eref.thieme.de/e-videos
*E-Videos* is an open access online section of the journal *Endoscopy* , reporting on interesting cases and new techniques in gastroenterological endoscopy. All papers include a high-quality video and are published with a Creative Commons CC-BY license. Endoscopy E-Videos qualify for HINARI discounts and waivers and eligibility is automatically checked during the submission process. We grant 100% waivers to articles whose corresponding authors are based in Group A countries and 50% waivers to those who are based in Group B countries as classified by Research4Life (see: https://www.research4life.org/access/eligibility/ ). This section has its own submission website at https://mc.manuscriptcentral.com/e-videos .

## References

[1] Tajima Y Nakanishi Y Tachimori Y Significance of involvement by squamous cell carcinoma of the ducts of esophageal submucosal glands. Analysis of 201 surgically resected superficial squamous cell carcinomas Cancer 2000 89 248 254 10.1002/1097-0142(20000715)89:2<248::aid-cncr7>3.0.co;2-q 10918152

[2] Wang WL Chang IW Moi SH Assessment of tumor extension to the ductal system of submucosal glands in patients with superficial esophageal squamous neoplasms: Implications for endoscopic resection J Thorac Cardiovasc Surg 2022 163 1951 1960 34649716 10.1016/j.jtcvs.2021.08.075

[3] Maida M Sferrazza S Murino A Effectiveness and safety of underwater techniques in gastrointestinal endoscopy: a comprehensive review of the literature Surg Endosc 2021 35 37 51 10.1007/s00464-020-07907-8 32856154

[4] Geng ZH Zhu Y Li QL Muscular injury as an independent risk factor for esophageal stenosis after endoscopic submucosal dissection of esophageal squamous cell cancer Gastrointest Endosc 2023 98 534 542 10.1016/j.gie.2023.05.046 37207844

